# Case Report: Toxocariasis as a rare trigger of vasculitis: a case-based review

**DOI:** 10.3389/fimmu.2026.1772680

**Published:** 2026-04-13

**Authors:** Sara Radovic, Aleksandra Plavsic, Rada Miskovic, Ana Drazic, Milos Todorovic, Irena Ostric Pavlovic, Uros Karic, Dragan Popovic, Marijan Micev, Snezana Arandjelovic

**Affiliations:** 1 Clinic for Allergy and Immunology, University Clinical Centre of Serbia, Belgrade, Serbia; 2 Faculty of Medicine, University of Belgrade, Belgrade, Serbia; 3 Clinic for Infectious and Tropical Diseases, University Clinical Centre of Serbia, Belgrade, Serbia; 4 Clinic for Gastroenterology and Hepatology, University Clinical Centre of Serbia, Belgrade, Serbia; 5 BeoLab Plus Medical Polyclinic, Belgrade, Serbia

**Keywords:** eosinophilia, infection-associated vasculitis, Toxocara cani(s), toxocariasis, vasculitis

## Abstract

**Introduction:**

Parasitic infections are increasingly recognized as potential triggers of autoimmunity and vasculitis, though evidence remains limited. *Toxocara canis (T.canis)*, one of the most prevalent helminthic infections worldwide, has been sporadically linked to autoimmune phenomena through mechanisms such as molecular mimicry, immune complex formation, and chronic inflammation.

**Case report and literature review:**

We describe a 45-year-old woman who developed histologically confirmed small-vessel vasculitis of the intestine with perforation, accompanied by cutaneous vasculitis, arthralgia, pruritus, eosinophilia, and elevated IgE and seropositivity for *T. canis*. Surgical resection with ileostomy led to spontaneous clinical remission without the need for immunosuppressive therapy. Immunologic evaluation revealed two coexisting organ-specific autoimmune diseases not typically associated with vasculitis: autoimmune thyroiditis (Hashimoto’s) and autoimmune cholangitis. We reviewed the available literature on *T. canis*-associated autoimmunity and vasculitis, summarizing clinical presentations and patient outcomes.

**Conclusion:**

Our report of histopathologically confirmed cases of small-vessel intestinal vasculitis associated with *T. canis*, leading to bowel perforation and remission without immunosuppression suggests that *T. canis* infection may represent an underrecognized trigger of autoimmunity and vasculitis. This case, together with a comprehensive review of the literature, indicates that *T. canis* infection should be considered in patients presenting with eosinophilia and elevated IgE, especially when accompanied by systemic or vasculitic manifestations.

## Introduction

Vasculitides represent a heterogeneous group of disorders characterized by inflammation of blood vessel walls, with diverse etiologies, clinical presentations, and prognoses. While the pathogenesis of primary systemic vasculitides (PSV) remains incompletely understood, infectious triggers have been increasingly recognized as important contributors to both the initiation and exacerbation of this disease (1, 2). Infection-associated vasculitis (IAV) can closely mimic PSV in clinical presentation, involving small, medium, or large vessels, and may lead to substantial diagnostic and therapeutic challenges. Timely distinction between IAV and PSV is critical, as management strategies differ considerably, particularly regarding the use of immunosuppressive therapy in the setting of active infection (2, 3).

Among the various infectious triggers, viral and bacterial pathogens have been the most extensively studied, whereas parasitic infections have received comparatively little attention despite their high global prevalence. Helminthic infections, in particular, are capable of inducing robust immune responses characterized by eosinophilia, elevated IgE levels, and Th2-skewed immune activation, features that may overlap with or precipitate autoimmune inflammation (2, 4). *Toxocara canis*, a common zoonotic roundworm of dogs, is one of the most widespread helminthic infections worldwide (5). Human exposure to toxocariasis is common in low- and middle-income countries, including many regions of Africa, where seroprevalence studies demonstrate widespread exposure and a substantial yet often underrecognized public health burden. These epidemiological data underscore the global relevance of toxocariasis as a potential trigger of immune-mediated complications (6, 7).

Human infection occurs when embryonated eggs from contaminated soil, food, or fomites are ingested, and after hatching in the gastrointestinal tract, the larvae penetrate the intestinal wall and enter the bloodstream. Clinical manifestations range from asymptomatic seropositivity to visceral (Visceral Larva Migrans - VLM) or ocular larva migrans, and in some cases, a common form marked by nonspecific symptoms such as fatigue, arthralgia, and pruritus (8, 9).

The potential relationship between *T. canis* infection and autoimmunity has been sporadically reported in the literature, with proposed mechanisms including molecular mimicry, cross-reactive epitopes, immune complex deposition, and breakdown of peripheral tolerance secondary to chronic inflammation (10). However, vasculitis as a complication of toxocariasis is exceptionally rare, with only a handful of cases described, and histopathologically confirmed intestinal vasculitis being even less common.

This article aims to (1) present a rare case of small-vessel vasculitis with intestinal perforation associated with *T. canis* infection (2) review the existing literature on the potential link between *T. canis* infection, vasculitis, and autoimmune diseases, with emphasis on proposed immunopathogenetic mechanisms, and (3) highlight the diagnostic challenges in differentiating infection-associated from primary vasculitis, underscoring the need for targeted diagnostic algorithms in patients presenting with eosinophilia and vasculitic features. A comprehensive literature search was performed using the MEDLINE/PubMed database with the keywords “vasculitis,” “autoimmunity,” “toxocariasis,” and “*T. canis*,” limited to articles and abstracts in English and French, without publication date limits.

## Case report

A 45-year-old woman was initially referred to an immunologist due to suspected vasculitis following surgery for bowel perforation. In the three months preceding the operation, she experienced loss of appetite and frequent diarrhea (3–4 stools per day, without mucus or blood). One month before surgery, she developed painful skin lesions on the lateral side of her right foot and the anterior surface of her left shin. These rapidly progressed to ulcerations that required surgical intervention. She subsequently presented to the emergency department with acute abdominal pain and vomiting, and was diagnosed with bowel perforation, necessitating bowel resection and temporary ileostomy formation.

**Figure 1:**
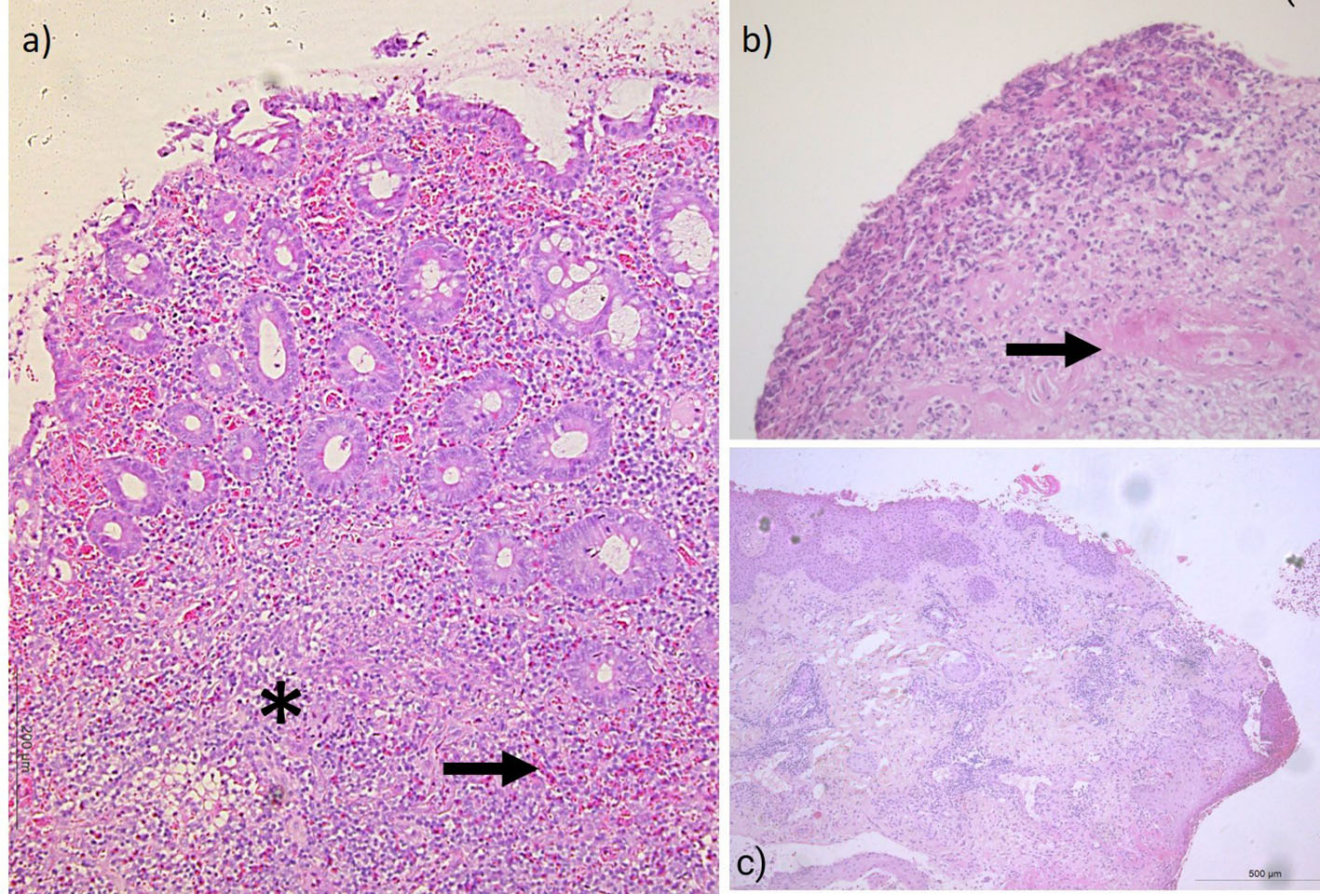
Histopathological findings demonstrate ulcerative and inflammatory changes in intestinal and cutaneous tissues, associated with vasculitis and prominent eosinophilic infiltration. **(A)** Erosive and inflammatory changes of intestinal mucosa and superficial submucosa with area of crypt epithelial destruction and vasculitis (asterisk) and foci with marked infiltration of eosinophiles (arrow) in deep portions (H&E, 20x). **(B)** Closer view of ulcerative and inflammatory changes with vascular wall fibrinoid necrosis (arrow) in some areas (H&E, 20x). **(C)** Cutaneous lesions with marginal area of deep dermal fissure-like ulceration (H&E, 5x).

Histopathological examination of the resected ileal tissue revealed inflammatory ulcers, subacute or chronic ileitis, fibro-inflammatory changes in the bowel wall, and diffuse moderate eosinophilic infiltrate (**Figure 1A**). Microfoci of perivasculitis and vasculitis were also observed (**Figure 1B**). A concurrent biopsy of a skin ulcer showed fibrinoid necrosis of small vessels and multiple foci of small-vessel vasculitis (**Figure 1C**).

The inflammatory infiltrate was predominantly lymphocytic, with fewer eosinophils and neutrophils. Corticosteroids were not initiated at the time, and the patient gradually recovered without immunosuppressive therapy. At this stage, she was treated at a regional general hospital.

At the initial evaluation by the immunologist, seven months after the first manifestation of vasculitis, a comprehensive personal and family history was obtained along with an extensive diagnostic workup. The patient resided in a rural area with a cattle farm and had kept a dog and a cat in the household until two years before symptom onset. She reported intermittent joint pain in recent months, with corresponding significant elevations in inflammatory markers on routine blood tests. She also reported generalized pruritus over the past two years, which had improved with over-the-counter antihistamines. Family history was positive for rheumatoid arthritis in the patient’s father. She had not travelled outside of Serbia.

**Figure 2:**
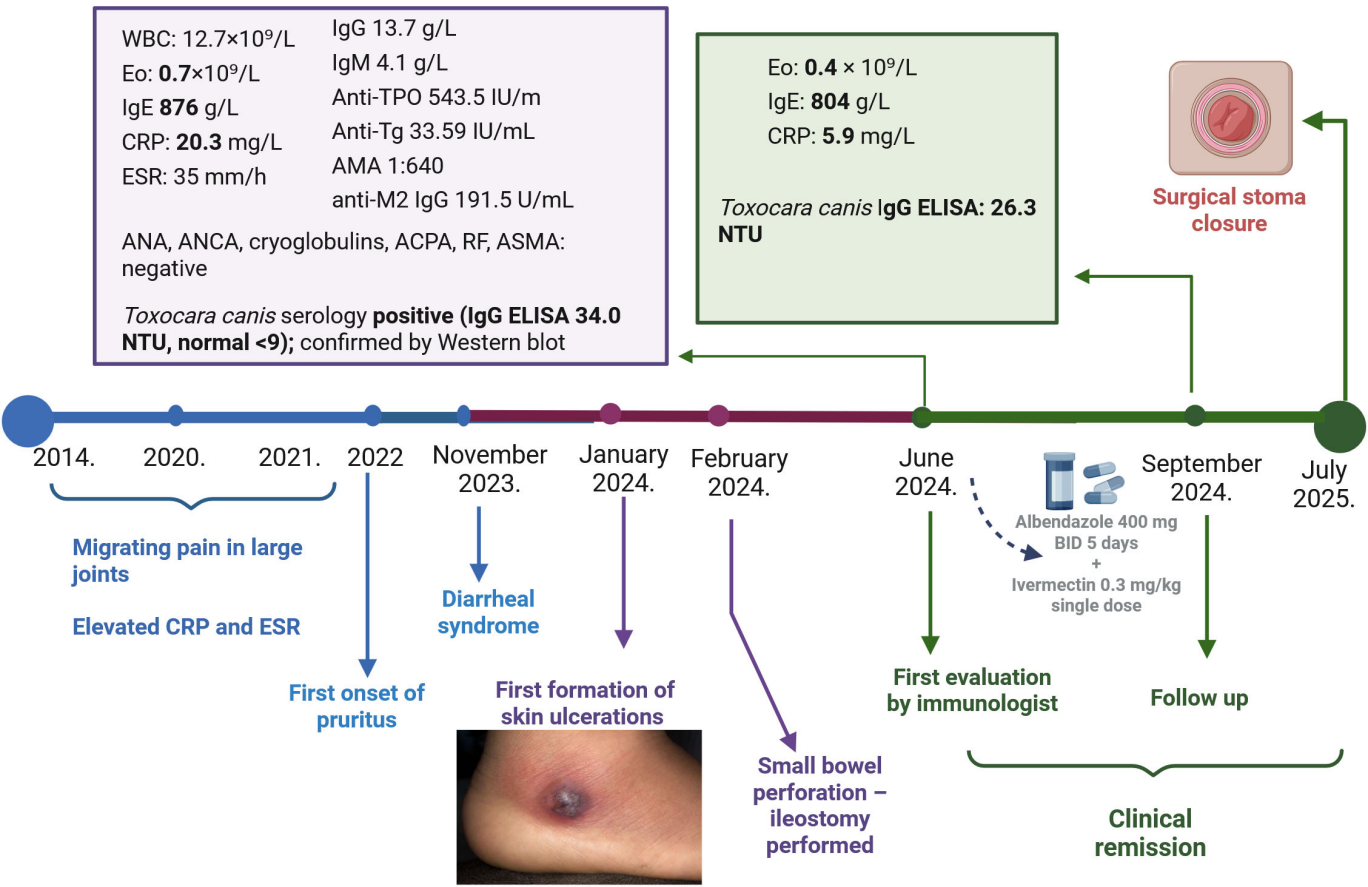
Timeline of clinical course and diagnostic findings. WBC, white blood cells; Eo, eosinophils; IgE, immunoglobulin E; IgG, immunoglobulin G; IgM, immunoglobulin M; CRP, C-reactive protein; SE, erythrocyte sedimentation; anti-TPO, anti-thyroid peroxidase antibodies; anti-TG, anti-thyroglobulin antibodies; AMA, anti-mitochondrial antibodies; ANA, anti-nuclear antibodies; ANCA, anti-neutrophil cytoplasmic antibodies; ACPA, anti-citrullinated protein antibodies; RF, rheumatoid factor; ASMA, anti-smooth muscle antibodies. Created in BioRender. Radovic, S. (2026) https://BioRender.com/dnraiy7.

Physical examination at the first immunological evaluation was unremarkable except for residual hyperpigmentation at the sites of previous skin ulcerations. Laboratory evaluation revealed eosinophilia (0.7×10^9^/L; ref. range: 0.03–0.35×10^9^/L, 7.30%, WBC: 8.9, ×10^9^/L), mildly elevated inflammatory markers (C-reactive protein (CRP) 20.3 mg/L, ref. range <5 mg/L, erythrocyte sedimentation rate (ESR) 35 mm/h, ref. range: 2–10 mm/h), and elevated cholestatic liver enzymes (alkaline phosphatase 207 U/L ref. range: 40–120 U/L, gamma-glutamyl transferase 105 U/L, ref. range <32 U/L). Immunological testing showed elevated IgM (4.1 g/L; ref. range: 0.4–2.3 g/L) and markedly elevated IgE (876 IU/mL; ref. range: 0–100 IU/mL). Autoantibody screening revealed markers of Hashimoto thyroiditis (positive anti-thyroid peroxidase (anti-TPO) antibodies and antithyroglobulin (anti-TG) antibodies) and primary biliary cholangitis (AMA titer 1:640, anti-mitochondrial M2 IgG 191.5 U/mL, ref. range <20 U/ml). Tests for ANA, ANCA, cryoglobulins, antiphospholipid antibodies, and complement levels were all negative (**Figure 2**).

Mesenteric CT angiography revealed no abnormalities in the vasculature. Chronic inflammatory changes were observed in the cecum, ascending and transverse colon, and in the terminal ileal segment entering the ileostomy, along with submucosal edema of the pyloric canal—consistent with ongoing inflammation. Transthoracic echocardiography was normal. Stool cultures and serologies for HIV, HBV, and HCV were negative. Allergy testing, including skin prick tests to inhalant and food allergens, together with the absence of atopic symptoms, excluded classical allergic causes of eosinophilia and elevated IgE.

Multiple stool samples were examined via microscopy by an experienced parasitologist (O&P including those done after the Kato-Katz concentration method) and no parasites were observed. However, serology for *T. canis* came back positive (IgG ELISA 34.0 NTU; ref.range <9 NTU). Western blotting was used to confirmed the presence of *T. canis*-specific IgG.

The patient was treated with albendazole (400 mg twice daily for 5 days) and a single dose of ivermectin (0.3 mg/kg), following consultation with an infectious disease specialist. As she was asymptomatic and showed no signs of active vasculitis, immunosuppressive therapy was not initiated. Ursodeoxycholic acid was initiated for autoimmune cholangitis, while no treatment was needed for Hashimoto thyroiditis due to euthyroid status. At three-month follow-up, eosinophil count decreased to 0.4 × 10^9^/L, CRP to 5.9 mg/L, while IgE remained elevated at 804 IU/mL. *T. canis* serology remained positive (IgG ELISA 26.3). A follow-up ileal biopsy performed through the ileostomy revealed histologic improvement with minimal, diffuse inflammation and no eosinophilic infiltration or evidence of vasculitis. Given the clinical improvement, ileostomy closure was recommended.

Based on the clinical presentation, histopathological findings, and serological results, the final diagnosis included small-vessel vasculitis, Hashimoto thyroiditis, and autoimmune cholangitis (AMA-positive primary biliary cholangitis). No recurrence of vasculitis was observed during subsequent follow-up.

### Patient perspective

Following surgical recovery and antiparasitic treatment, the patient reported substantial improvement in symptoms and quality of life. She expressed dissatisfaction with the temporary ileostomy but remained strongly motivated to undergo surgical restoration of bowel continuity once clinically appropriate.

## Discussion and review of the literature

Although it is well established in the literature that parasites can serve as a trigger factor for autoimmunity, with autoantibodies found in the sera of patients with Chagas’ disease, malaria, leishmaniosis, schistosomiasis, and onchocerciasis, only two studies have investigated the presence of autoantibodies in patients with toxocariasis (10). *Kaya* et al. reported a statistically significant difference in overall autoantibody positivity between symptomatic patients with *T*. *canis* infection confirmed by ELISA and control groups, with 45% of patients testing positive. The most frequently detected autoantibody was ASMA, followed by AMA and anti-dsDNA (11). Similarly, *Obwaller* et al. demonstrated a marked increase in speckled staining pattern in the immunofluorescence test and with anti-snRNP reactivity in the immunoblot assay among symptomatic patients with *T.canis* infection, compared to both asymptomatic individuals and controls, concluding that there may be a potential correlation between elevated autoantibody levels and the presence of clinical inflammation (12).

**Figure 3:**
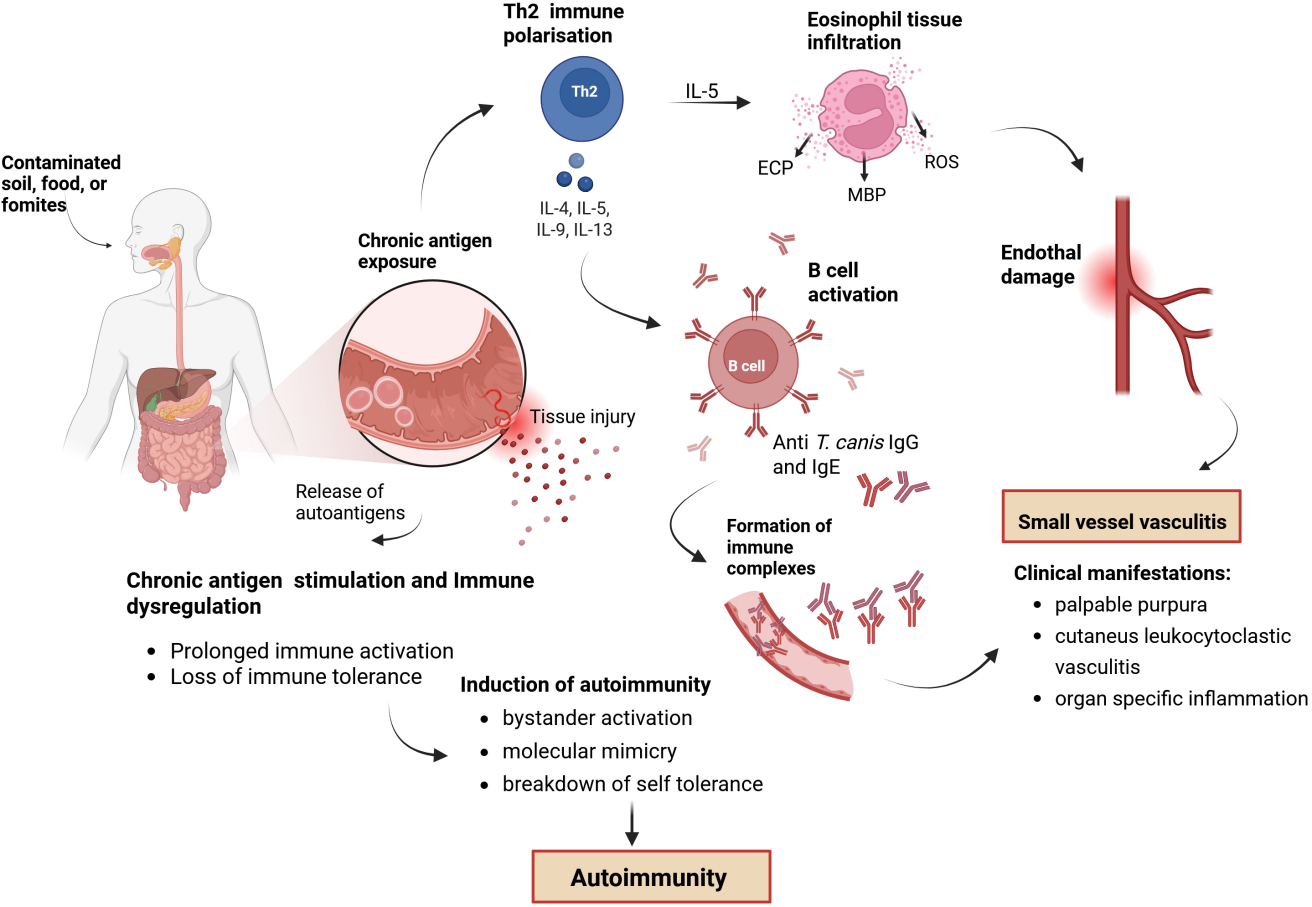
Proposed pathogenetic model linking toxocariasis, immune dysregulation, and secondary vasculitis. Infection via contaminated soil, food, or fomites leads to chronic antigen exposure and tissue injury, releasing autoantigens. Th2 immune polarization and eosinophil infiltration contribute to endothelial damage, while B cell activation induces the production of anti-Toxocara IgG and IgE antibodies. Formation of immune complexes and mechanisms such as bystander activation and molecular mimicry promote autoimmunity. These combined processes result in small-vessel vasculitis, with clinical manifestations including palpable purpura, cutaneous leukocytoclastic vasculitis, and organ-specific inflammation. Created in BioRender. Radovic, S. (2026) https://BioRender.com/zepw36k.

The exact pathomechanism causing autoantibody production in toxocariasis is still not well understood. It is hypothesized that tissue injury during larval migration may result in the release of autoantigens, and/or that molecular mimicry between parasitic and host antigens contributes to the development of an autoimmune response (12). Potential cross-reactive epitopes were found between thyroid peroxidase and peroxidasin-like proteins in *T. canis* supporting the molecular mimicry theory, but no *in vivo* studies have been yet performed to confirm the correlation (13). Although specific autoantibodies have been detected, we found no studies or publications exploring the relationship between *T. canis* infection and organ-specific autoimmune diseases as presented in our patient with positive anti-TPO and anti-TG antibodies. Another potential underlying mechanism involves the formation of immune complexes, specifically IgE/anti-IgE complexes, potentially triggering a type III hypersensitivity reaction and subsequent autoimmune inflammation (14) (**Figure 3**).

Our literature search identified 12 cases of vasculitis associated with *T. canis* infection and one case of systemic lupus erythematosus attributed to toxocariasis (15–27). Cerebral vasculitis represented the most common subtype while leukocytoclastic vasculitis, Henoch-Schönlein purpura and Churg-Strauss syndrome each occurred in 2 out of 12 cases (**Table 1**). A key problem in observed literature was the criteria used to classify vasculitis and the frequent absence of histopathological confirmation. Hypereosinophilia and high levels of IgE prompted parasitosis workup in most cases, although the diagnosis of *toxocariasis* was usually ultimately confirmed using serological testing (ELISA), the current gold standard for diagnosis. Only in the first described case by *Krauss* et al. (15) the diagnosis followed a biopsy of panniculitis that revealed larvae of *T. canis.*

**Table 1:** Summary of published cases of Toxocara related vasculitis.

	Type of vasculitis	Age/Gender	Diagnosis of T.canis	Clinical characteristics	Treatment	Outcome	Author/year
1.	Leukocytoclastic vasculitis	F/36	Panniculitis containing larvae of T. canis	Skin lesions, migratory, pruritus, hepatomegaly, cough and panniculitis	Prednisone (15 mg/day)	Recovery	Krauss, 1995 (15)
2.	Churg-Strauss syndrome	F/48	Positive serology (ELISA) for T.canis	Late-onset asthma, multineuritis, blood and pulmonary eosinophilia, positive p-ANCA	Albendazole and prednisone	Recovery	Raschilas, 1996 (16)
3.	Henoch-Schönlein purpura	M/17	Positive anti-Toxocara IgG and IgE (ELISA) and positive Western blot test	Palpable purpura, oligoarthritis, acute abdominal pain, microhematuria, hypereosinophilia with positive ANA	None	Recovery with transient presence of ANA	Hamidou, 1999 (17)
4.	Systemic vasculitis with lymphocytic temporal arteritis	F/66	Positive anti-Toxocara IgG and IgE (ELISA) and positive Western blot test	Fever, pulmonary infiltrates, skin lesions, sinusitis, hypereosinophilia, abnormalities of liver function, and renal involvement, positive ANA. Bilateral temporal artery biopsy results showed lymphocytic vasculitis.	None	Recovery	Hamidou, 2002 (18)
5.	Churg-Strauss syndrome	F/74	Positive anti-Toxocara IgG (ELISA)	Fever, nodular eruption, eosinophilic cellutis, myalgia, pleurisy and eosinophilic pneumonia	Prednisone and thiabendazole	Recovery	Lhote, 2004 (19)
6.	Leukocytoclastic vasculitis	M/51	Positive anti-Toxocara IgG (ELISA) and positive Western blot test	Skin rash, hypereosinophila, chornic eycema, hyperIgE	Ivermectine 200mg/kg, flubendazole 200mg/day for 3 days and prednisolon	Recovery	Attout, 2004 (20)
7.	Cerebral vasculitis	F/75	Positive anti-Toxocara IgG (ELISA) and positive Western blot test in serum and in CSF	Fever, weight loss, right cerebellar signs and symptoms	Albendazole (800 mg/ for 4 weeks) and dexamethason (slowly tapering off within 3 weeks)	Recovery	Helbok, 2006 (21)
8.	Henoch–Schönlein purpura	28/F	Positive anti-Toxocara IgG (ELISA) and positive Western blot test	Fever, abdominal pain, palpable purpuric rash, inflammatory arthralgia and proteinuria over 2g/24h	Diethylcarbamazine and corticosteroid therapy for one year	Recovery	Bellanger, 2011 (22)
9.	Cerebral vasculitis	49/M	Positive anti-Toxocara IgG and IgE (ELISA) and positive Western blot test	Infarction of the right ACA, syndrome of callosal dysconnection (unilateral left agraphia and apraxia), apathy, mutism hypereosinophilia, hypergammaglobulinemia, an inflammatory syndrome. High IgE in CSF. Latent tertiary syphilis without nerve damage	Albendazole (800 mg-day over 5 days) and corticosteroid therapy (70 mg per day) for one month, and an antisyphilitic therapy with Doxicycline (400 mg per day over 14 days)	Progression of cerebral vasculitis caused by T. canis, despite the treatment after 5 months	Lompo, 2012 (23)
10	Retinal vasculitis	16/M	Positive anti-Toxocara IgG (ELISA)	Left eye pain, floaters, decreased visual acuity (4/200) and hypereosinophilia. The diagnostic workup was conclusive for neuroretinitis and retinal vasculitis.	Oral prednisone albendazole.	Seventeen months after his initial presentation, his visual acuity improved to 20/80.	Besirli, 2013 (24)
11	Leukocytoclastic vasculitis with IgA deposits	64/M	Positive anti-Toxocara IgG (ELISA) and positive Western blot test	Hypereosinophilia, fever, abdominal pain, arthralgia, purpuric rash, increased inflammatory parameters, hypergammaglobulinemia positive anti-C1q antibodies, ANA, dsDNA RF. Low C3, C4, and CH50	Albendazole 15 days with corticosteroids tapering (for 4 months)	Recovery. Low titer of ANA persisted	Boysson, 2015 (25)
12	Cerebral vasculitis	56/F	Positive anti-Toxocara IgG (ELISA)	Hypereosinophilia, elevated IgE, weakness in right arm, dizziness, chest pain, eosinophilic myocarditis and cerebral vasculitis.	Prednisolone 500 mg/day for 3 days followed by 50 mg/day tapering and albendazole (1000 mg/day)	Recovery.	Chatzikonstantinous, 2022 (26)
13	Systemic vasculitis with cutaneous manifestation and bowel perforation	45/F	Positive anti-Toxocara IgG (ELISA) and positive Western blot test	Arthragia, fatigue, cutaneous vasculitis, small bowel vasculitis with perforation, hypereosinophilia, elevated IgE, positive AMA M2 and antithyroid antibodies	None Surgical therapy	Recovery. Simultaneous diagnoses of autoimmune cholangitis and autoimmune thyroiditis	Radovic S et al 2025 [Our case]

*p-ANCA- perinuclear anti-neutrophil cytoplasmic antibodies; ANA- antinuclear antibody; IgE- immunoglobulin E; IgG- immunoglobulin G; ACA-anterior cerebral artery; CSF- cerebrospinal fluid; RF- rheumatoid factor; C3- complement component 3; C4- complement component 4; dsDNA- anti-double stranded DNA antibody; AMA M2- anti-mitochondrial M2 antibody WB.

The final row summarizes the present series of 11 patients.

Autoantibodies were found in four patients, with ANA being the most common (3/12) (17, 18, 25). The variation in antibody detection may be explained by variability in test availability and the diagnostic workup performed. While presence of elevated inflammatory markers is common in both VLM syndrome and vasculitis, it is not a hallmark of common/covert toxocariasis (28). This can cause a diagnostic problem, because both ESR and CRP are nonspecific markers and their elevated levels cannot reliably distinguish between autoimmune inflammation in vasculitis and inflammation caused by coexisting infection (29).

Although data on infection duration and autoimmune induction are lacking, our patient’s prolonged animal exposure suggests chronic antigenic stimulation by *T. canis*. In retrospect, it is likely that our patient had a chronic, subclinical *T. canis* infection for years, as suggested by longstanding nonspecific symptoms such as arthralgia, pruritus, and elevated inflammatory markers. The subsequent development of vasculitis may have been triggered by a shift in immune homeostasis, possibly due to a transient decline in immune control, allowing for unrestrained antigenic stimulation.

The clinical manifestations and the presentation of vasculitis varied but in most cases constitutional symptoms were present along with skin manifestations (7/12). Interestingly, none of the patients exhibited an urticarial rash, which is typically the most common skin manifestation (30). Our patient had prodromal symptoms of loss of appetite and chronic diarrhea and pruritus, all common presentations of toxocariasis (31). She also experienced arthralgias, which are not typically associated with the organ-specific autoimmune diseases she was later diagnosed with, autoimmune cholangitis and Hashimoto’s thyroiditis, but have been commonly documented in patients with toxocariasis (32).

What sets our case apart from previously reported ones, and highlights its rarity, is the histopathological confirmation of small intestinal vasculitis with eosinophilic infiltration and subsequent perforation in the context of *T. canis* infection. Following the confirmation of vasculitis in both, the skin and intestinal biopsy no immunosuppressive therapy was started at the time, considering the patient was treated in a secondary medical center with only occasional opportunities for immunologist consultation. At that time, no imaging studies were performed, including an angiography, which significantly limits the assessment of vasculitic changes in the mesenteric blood vessels. This is an important gap, since radiographic methods are essential in the initial diagnostic workup and evaluation of suspected intestinal vasculitis (33). In our case, the first mesenteric angiography was performed seven months after the initial presentation of vasculitis.

Interestingly, spontaneous regression of vasculitis symptoms, arthralgia, and general malaise was observed after the surgical treatment, prior to the use of antiparasitic therapy. This occurrence may be explained by the potential elimination of a local source of chronic antigenic stimulation, as histopathological examination of the resected ileal segment revealed features of subacute/chronic ileitis with eosinophilic infiltration and microfoci of vasculitis, suggesting a possible localization of parasitic antigens within the affected ileal segment. Resection of the affected segment, along with temporary diversion of the remaining gastrointestinal tract via ileostomy, may have led to a reduction in systemic exposure to parasitic antigens, thereby interrupting the ongoing immune stimulation. This mechanism could account for the partial or complete spontaneous remission of inflammatory and autoimmune manifestations prior to pharmacologic intervention. While no direct evidence supports spontaneous vasculitis remission following bowel resection and ileostomy in parasitic infection, analogous mechanisms are well-documented in inflammatory bowel disease (34, 35). The chronological association between surgical intervention and clinical improvement raises the question of whether, in patients with VLM and prominent inflammatory manifestations, surgery may offer not only mechanical relief but also help in resolving the immune-mediated process.

The proposed pathogenetic mechanisms among Toxocariasis, vasculitis, and autoimmunity remain hypothetical and require further experimental validation.

In the reviewed literature, we identified only two cases of *T.canis* associated vasculitis in which therapy was not initiated; fortunately, both resulted in spontaneous recovery (17, 18). The remaining patients were treated with antiparasitic agents and glucocorticoids, leading to full recovery in all but one male patient, who showed progression of cerebral vasculitis despite treatment during a five-month follow-up (23). The most commonly used antiparasitic agents for treating *T. canis* infection are benzimidazole derivatives, particularly albendazole, due to its pharmacokinetic properties and widespread accessibility (36). Our decision to simultaneously use ivermectin, which is not a standard treatment option for toxocariasis, was prompted by the high epidemiological risk for other parasitic infection and limited diagnostic resources. There remains considerable uncertainty regarding the efficacy of available anthelmintic therapies due to a lack of robust prospective controlled trials. In order to assess the therapeutic response, clinical monitoring is most valuable. It is recommended that the optimal follow up time be one month for common toxocariasis or 3 months for more severe forms (34). Longer follow-up periods without preventive measures may result in misinterpreting reinfection as therapeutic failure (37). Eosinophilic count is a helpful biomarker in determining therapeutic outcome. Depending on the parasitic load, eosinophil levels typically rise rapidly within the first week of treatment due to *T. canis* lysis and generally return to normal within one month while IgE levels decline more slowly, resolving progressively within three months post-treatment. On the contrary, IgG testing by ELISA and Western blot does not appear suitable for post-treatment follow-up because of the heterogeneous results of its kinetics that suggest a much slower decline (38). In some cases, the ELISA test remained positive for up to four years following treatment (39).

The decision to close the ileostomy was based on the absence of clinical relapse, normalization of eosinophil counts, clear visualization of mesenteric vessels on MSCT angiography, and histological improvement.

This report is limited by its single-case design, which precludes generalization and definitive establishment of a causal link between *T. canis* infection and vasculitis. The immunopathogenic mechanisms discussed are based on literature review and clinical inference rather than direct experimental evidence or longitudinal immune monitoring in the patient. Additionally, the initial diagnostic workup lacked advanced imaging such as mesenteric angiography, which may have limited comprehensive assessment of vascular involvement. The coexistence of other autoimmune conditions, namely Hashimoto’s thyroiditis and autoimmune cholangitis, further complicates attribution of vasculitis solely to *T. canis* infection. The existence of these two organ-specific autoimmune diseases, indicates an autoimmune predisposition and an imbalance of immunoregulation that may be associated with an increased tendency toward aberrant immune responses to infection. Under these circumstances, vasculitis may be interpreted as a secondary process, potentially precipitated by the immune response to toxocariasis, while the autoimmune diseases represent parallel manifestations of immune predisposition. Finally, the relatively short follow-up period restricts evaluation of long-term disease course and relapse risk. Future studies with larger cohorts and longer follow-up are necessary to better define the relationship between toxocariasis and vasculitis. Nevertheless, this case is significant as it provides one of the few histopathologically confirmed examples of small-vessel vasculitis with intestinal perforation linked to *T. canis*, thereby expanding the clinical spectrum of infection-associated vasculitis. It underscores the importance of considering parasitic infections in the differential diagnosis of vasculitis, especially in patients presenting with eosinophilia and elevated IgE, and highlights the need for multidisciplinary diagnostic approaches. This contribution may aid clinicians in earlier recognition and tailored management of similar cases, ultimately improving patient outcomes.

## Conclusion

Parasitic infection should be considered in patients with eosinophilia, elevated IgE, and vasculitic features to avoid misclassification as primary vasculitis. Our report contributes to the limited but growing evidence linking *T. canis* to autoimmunity and vasculitis, emphasizing the need for further studies to elucidate underlying mechanisms and optimal treatment strategies.

## Data Availability

The raw data supporting the conclusions of this article will be made available by the authors, without undue reservation.
